# One-Step Reverse-Transcription FRET-PCR for Differential Detection of Five *Ebolavirus* Species

**DOI:** 10.1371/journal.pone.0126281

**Published:** 2015-05-27

**Authors:** Guangwu Lu, Jilei Zhang, Chuntao Zhang, Xiaolu Li, Dawei Shi, Zhaopeng Yang, Chengming Wang

**Affiliations:** 1 Jiangsu Co-innovation Center for Prevention and Control of Important Animal Infectious Diseases and Zoonoses, Yangzhou University College of Veterinary Medicine, Yangzhou, Jiangsu, 225009, P. R. China; 2 National Institute for Food and Drug Control (NIFDC), China Food and Drug Administration, Chongwen District, Beijing, 100050, P. R. China; 3 College of Chemistry, Chemical Engineering and Biotechnology, Donghua University, Shanghai, 201620, P. R. China; Division of Clinical Research, UNITED STATES

## Abstract

Ebola is an emerging infectious disease caused by a deadly virus belonging to the family Filoviridae, genus *Ebolavirus*. Based on their geographical distribution, *Ebolavirus* has been classified into total five species so far, mainly *Zaire*, *Sudan*, *Taï Forest*, *Bundibugyo* and *Reston*. It is important to be able to differentiate the *Ebolavirus* species as they significantly differ in pathogenicity and more than one species can be present in an area. We have developed a one-step step-down RT-PCR detecting all five *Ebolavirus* species with high sensitivity (1 copy of *Ebolavirus* DNA, 10 copies of RNA and 320 copies of RNA spiked in 1 ml whole blood). The primers and FRET-probes we designed enabled us to differentiate five *Ebolavirus* species by distinct *T*_m_ (*Zaire*: flat peaks between 53.0°C and 56.9°C; *Sudan*: 51.6°C; *Reston*: flat peaks between 47.5°C and 54.9°C; *Tai Forest*: 52.8°C; *Bundibugyo*: dual peaks at 48.9°C and 53.5°C), and by different amplicon sizes (*Zaire* 255bp, *Sudan* 211bp, *Reston* 192bp, *Taï Forest* 166bp, *Bundibugyo* 146bp). This one-size-fit-all assay enables the rapid detection and discrimination of the five *Ebolavirus* species in a single reaction.

## Introduction

Ebola Virus Disease (EVD), previously known as Ebola hemorrhagic fever, is an emerging infectious disease caused by a deadly virus of the family Filoviridae, genus *Ebolavirus* [1, 2]. Of the five identified *Ebolavirus* species, four of them (*Zaire ebolavirus*, *Sudan ebolavirus*, *Taï Forest ebolavirus* and *Bundibugyo ebolavirus*) are known to cause disease in humans, whereas the fifth (*Reston ebolavirus*) causes disease in non-human primates and swine only [3–6]. Since human infections were first reported in 1976 on the Ebola River in Democratic Republic of the Congo [7], outbreaks of EVD have occurred into twelve countries (South Sudan, Democratic Republic of Congo, Côte d’Ivoire, Gabon, Uganda, Republic of Congo, Guinea, Philippines, Nigeria, Senegal, Liberia, Mali). There have also been imported cases in Sierra Leone, Spain, and the United States of America [8–10, 3, 11, 12]. By January 25, 2015, a total of 22, 092 cases of Ebola infections have been confirmed, of which 8, 810 cases of EVD have been fatal [12].

While simple or multiplex PCRs have been successfully used to detect *Ebolavirus*, most of the published assays can only detect a single *Ebolavirus* species [13, 14] or, in the case of those that detect multiple *Ebolavirus* species, they show reduced sensitivity [15–17]. Whereas simple PCR tends to provide high sensitivity but lacks ability to differentiate multiple targets simultaneously [13, 14, 18], multiplex PCR has the capacity to detect and differentiate multiple targets but tends to have a reduced sensitivity [17, 19–21]. The TaqMan RT-PCR assay targeting the nucleoprotein gene of *Zaire ebolavirus* developed by Huang Y et al. [14] had a wide range in sensitivity, from 10^3^ to 10^9^ virus copies per reaction. The primers and fluorogenic probe of the one-step qRT-PCR described by Towner JS et al. [13] were specific for *Sudan ebolavirus* and showed a high sensitivity (10^3^ genomic-sense *Ebolavirus*-RNA copies per ml; one genomic-sense RNA per reaction). Panning et al. [19] established a complicated system with 5 primers and 3 probes for Filoviridae with the detection limit from 487 to 4546 RNA copies/ml depending on the species of *Ebolavirus*. Trombley et al. [20] established a system to detect multiple hemorrhagic fever viruses including *Ebolavirus* by using 48 TaqMan-based PCR assays with the limit of detection for the assays ranging from 10 to 0.001 plaque-forming units / PCR.

While RT-PCR with high sensitivity and specificity have been widely used to quantify RNA viruses, RNA templates as positive controls were not always available and safe for highly infective pathogens such as SARS virus and highly pathogenic avian influenza viruses. In addition, RNA templates from killed viruses tend to degrade under the influence of RNase and metal ions. In comparison, the VLPs equipped with specific RNA fragments have been widely used as stable, reliable and safe positive controls for RT-PCR [22–24].

A generic PCR that can detect and differentiate all *Ebolavirus* species is needed as increased international movement of people increases the potential of transmission for multiple *Ebolavirus* species with varying pathogenicity [11, 17, 25–28]. Here we report a highly-sensitive one-step reverse-transcription FRET-PCR capable of rapidly identifying and differentiating all five *Ebolavirus* species.

## Materials and Methods

### Plasmids containing portion of *Ebolavirus*

Five plasmids, each compromised the first 600 bp (5’end) of five *Ebolavirus* species (*Zaire ebolavirus*, *Sudan ebolavirus*, *Reston ebolavirus*, *Taï Forest ebolavirus* and *Bundibugyo ebolavirus*), were created (GenScript, Nanjing, China) to serve as positive controls and quantitative standards in this study. The nucleotide fragments were synthesized and inserted into the pUC57 cloning vector, and the resulting plasmids were linearized with *Sac* I (Takara Biotechnology, Dalian, China) and quantified using the PicoGreen DNA fluorescence assay (Molecular Probes, Eugene, OR, USA) for preparation of quantitative standards (10^4^, 10^3^, 10^2^, 10^1^, 10^0^ copies /10 μl).

### Preparation of *Ebolavirus* RNA transcripts

Since it is not allowed to use viable *Ebolavirus* for research in China and none of five *Ebolavirus* species was available to us, transcripts made from the plasmids described above and virus-like-particles (VLPs) containing *Ebolavirus* RNA fragment were used as positive controls. The transcription reaction was run with MEGAscript Kit (Ambion by life technologies, Carlsbad, CA, USA) according to the manufacturer’s instructions. After incubation at 37°C for 4h, the transcribed products were treated with TURBO DNase (Ambion by life technologies, California, USA), followed by precipitation with lithium chloride. Finally, the yield of RNA transcripts was determined with the Quant-iT RiboGreen RNA Kit (Molecular Probes, Eugene, OR, USA) for preparation of the RNA standards (10^5^, 10^4^, 10^3^, 10^2^, 10^1^, 10^0^copies /10μl).

### Construction and expression of virus-like particles (VLPs) containing *Ebolavirus* NP RNA

In addition to the positive controls of plasmids and transcripts described above, we prepared also the VLPs containing the *Ebolavirus* NP RNA fragment to further verify the specificity and reverse-transcription efficiency of the established PCR in this study. The MS2 VLPs containing the NP RNA fragments of each of these five *Ebolavirus* species were prepared as the method described by Drosten *et al*. [29]. The primers (forward primer: 5’-ATGAATTCTCCTGCTCAACTTCCTGTCG-3’; reverse primer: 5’-GCAAGCTTGTTAGTAGATGCCGGAGTTT-3’) of MS2 phage (ATCC 15591-B1) assembly protein gene, coat protein gene and the fragment of *Eblolavirus* RNA NP gene was synthesized according to their sequences from GenBank data (Gene Accession#: KC24280) and were used to amplify their cDNAs by RT-PCR. Expression vector pTrc99a (Pharmacia) and their cDNA fragments were ligated with T4 DNA ligase after digestion with restriction enzyme. The prokaryotic expression was obtained by transformation of recombinant plasmids into *E*. *coli* JM109.The obtained VLPs were purified and tested by quantitative analysis and RT-PCR.

### Inoculation of transcribed *Ebolavirus* RNA and VLPs in human blood

Ten μl serially diluted transcribed RNAs and VLPs (10^6^, 10^5^, 10^4^, 10^3^, 10^2^, 10^1^ copies/10 μl) were thoroughly mixed with 50 μl human whole blood (collected in EDTA) and 150 μl 1× PBS for RNA extraction (High Pure RNA Isolation Kit, Roche, Mannheim, Germany). After vortexing for 15s with Lysis/-Binding Buffer and Red Cell Lysis Buffer (Roche Diagnostics GmbH, Mannheim, Germany), the blood samples were transferred to the High Pure filter tube and RNA eluted with 60 μl elution buffer which was stored at -80°C until thawed at room temperature for RT-PCR.

### One-step FRET RT-PCR for *Ebolavirus*

#### Primers and probes

**Fig 1:**
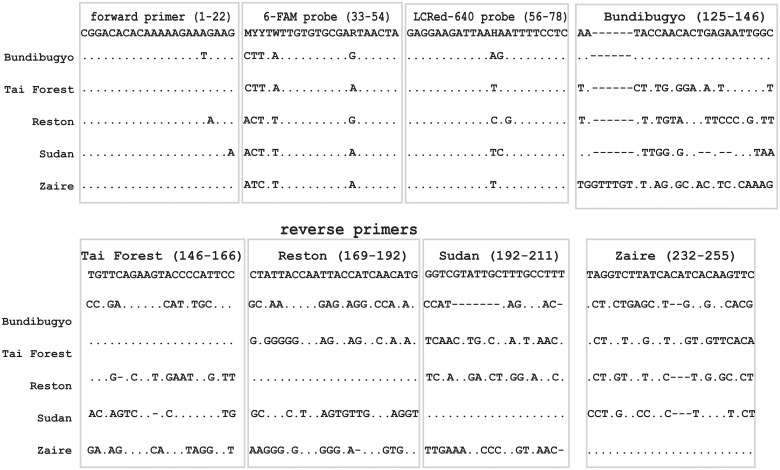
Alignment of primers and probes for five *Ebolavirus* species. Nucleotide sequences of the primers (one forward primer and five reverse primers for all *Ebolavirus* species) and probes are shown at the top of the frames while the corresponding nucleotide sequences of five species of *Ebolavirus* are shown at the bottom. Dots indicated that nucleotides are identical to those of primers/probes, and strips indicated deletion of nucleotides. The upstream primer was used as shown while the downstream primers and probes were used as reverse oligonucleotides. The 6-FAM and LCRed-640 probes had 5 and 1 degenerate nucleotides, respectively. The numbers associated with primers/probes indicate the positions of primers/probes in the whole genome of *Ebolavirus*.

The full genome sequences of five *Ebolavirus* species (two representing GenBank Accession # for *Zaire ebolavirus* NC_002549, KC24280; *Sudan ebolavirus* NC_006432, KC54539; *Reston ebolavirus* NC_004161, JX477166; *Taï Forest ebolavirus* NC_014372, FJ217162; *Bundibugyo ebolavirus* NC_014373, KC545396), and those two monophyletic genus (*Marburgvirus* KC545388, NC_001608; *Cuevavirus* JF828358, NC_016144) were obtained from GenBank (Fig 1). Based on the conserved and variable regions determined by Clustal Multiple Alignment, we identified highly conserved regions in 1–146 bp of the genome which were common to all five *Ebolavirus* species and used for the forward primer, 6-FAM probe and LCRed 640 probe (Fig 1, Table 1). The regions between 146 bp and 255 bp were highly conserved for individual *Ebolavirus* species but were highly variable among the different species and were thus used to design five reverse primers to specifically amplify only a single *Ebolavirus* species (Fig 1, Table 1). The primers and probes demonstrated high level of mismatches with *Cuevavirus* (35–40 mismatches) and *Marburgvirus* (no corresponding fragment).

**Table 1:** Primers and probes used in this study.

Primer/probe	Sequence (5’→3’)	Location	Note
Forward primer	CGGACACACAAAAAGAAAGAAG-3’	1–22	Common to all *Ebolavirus* species
6-FAM probe	6-FAM- TAGTTAYTCGCACACAAWARRK-phos	33–54	
LCRed probe	GAGGAAAATTDTTAATCTTCCTC-LCRed640	56–78	
Reverse primer-1	GCCAATTCTCAGTGTTGGTATT	125–146	Specific for *Bundibugyo*
Reverse primer-2	GGAATGGGGTACTTCTGAACA	146–166	Specific for *Taï Forest*
Reverse primer-3	CATGTTGATGGTAATTGGTAATAG	169–192	Specific for *Reston*
Reverse primer-4	AAAGGCAAAGCAATACGACC	192–211	Specific for *Sudan*
Reverse primer-5	GAACTTGTGATGTGATAAGACCTA	232–255	Specific for *Zaire*

#### Thermal cycling

One-step RT-PCR was performed in a LightCycler 480 II real-time PCR platform. Each reaction was performed in a 20μl final volume containing 10μl of plasmid DNA, transcribed RNA or VLPs and 10μl master mix with a final concentration of 1μM forward primer, 0.2μM 6-FAM probe, 0.2μM LCRed 640 probe, 1μM Reston ebolavirus reverse primer and 0.5μM reverse primers for the four other *Ebolavirus* species (Table 1). For each sample in 20μl reaction, 0.14 U SuperScript III reverse transcriptase (Invitrogen) was added and other reaction components were used as described [30]. Thermal cycling consisted of one reverse transcription step, 18 high-stringency step-down cycles and 30 relaxed-stringency fluorescence acquisition cycles. The reverse transcription step was at 55°C for 15 min, followed by denaturation at 95°C for 2 min. The 18 high-stringency step-down thermal cycles were 6×1 sec @ 95°C, 12 sec @ 70°C, 8 sec @ 72°C; 9×1 sec @ 95°C, 12 sec @ 68°C, 8 sec @ 72°C; 3×1 sec @ 95°C, 12 sec @ 66°C, 8 sec @ 72°C. The relaxed-stringency fluorescence acquisition cycling consisted of 30×1 sec @ 95°C, 8 sec @ 52°C, 30 sec @ 67°C and 30 sec @ 72°C.

#### High-resolution melting genotyping analysis

**Fig 2:**
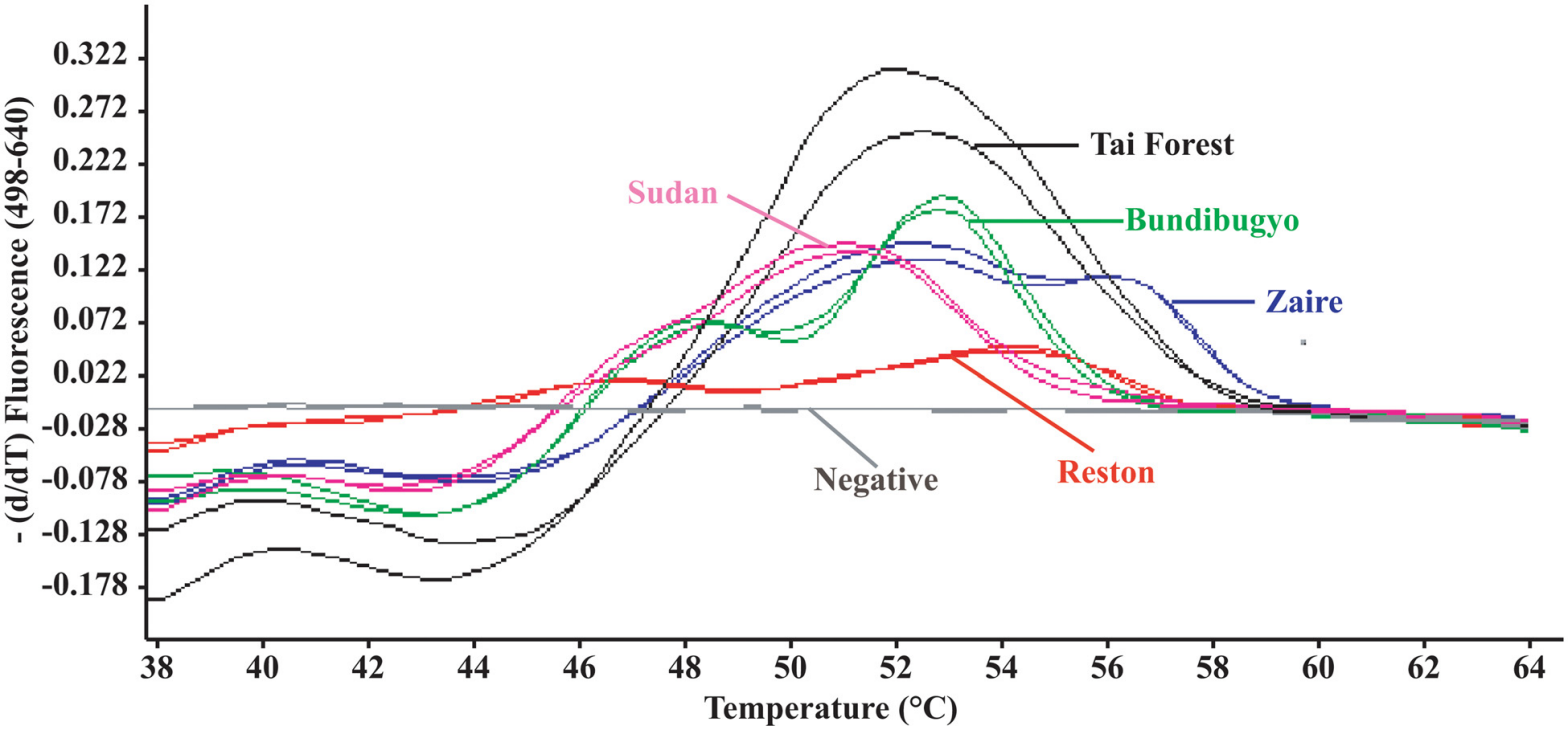
Discrimination of five *Ebolavirus* species through HRM genotyping analysis. Following the completion of PCR, the *T*_m_ of probe hybridization to the targets was determined by high-resolution melting (HRM) curve analysis as the peak of the second derivative of the fluorescence released during a temperature increase from 38°C to 85°C. Fluorescence F4/F1 demonstrates unique and distinct *T*_m_ differences between *Zaire ebolavirus* (flat dual peaks between 53.0°C and 56.9°C, blue), *Sudan ebolavirus*, (51.6°C, pink), *Reston ebolavirus* (flat dual peaks between 47.5°C and 54.9°C, red), *Taï Forest ebolavirus* (52.8°C, black), *Bundibugyo ebolavirus* (dual peaks at 48.9°C and 53.5°C, green), and negative control (no melting curve, grey). When two concentrations of the targets (100 and 10 copies of the gene / 20 μl reaction system) were used for each *Ebolavirus* species, the peaks and shapes of the melting curves were the same. Triplicates were performed to show the ranges at which the FRET gave melting temperatures.

After the FRET-PCR was completed, the melting curve analysis for probes annealing to the PCR products was determined by monitoring the fluorescence from 38°C to 85°C as described previously [30, 31]. Data were analyzed as 640 nm: 530 nm (F4/F1) fluorescence ratios, and the first derivative of F4/F1 (-d(F4/F1)/dt) was evaluated (Fig 2).

#### Specificity of the one-step RT FRET-PCR

**Fig 3:**
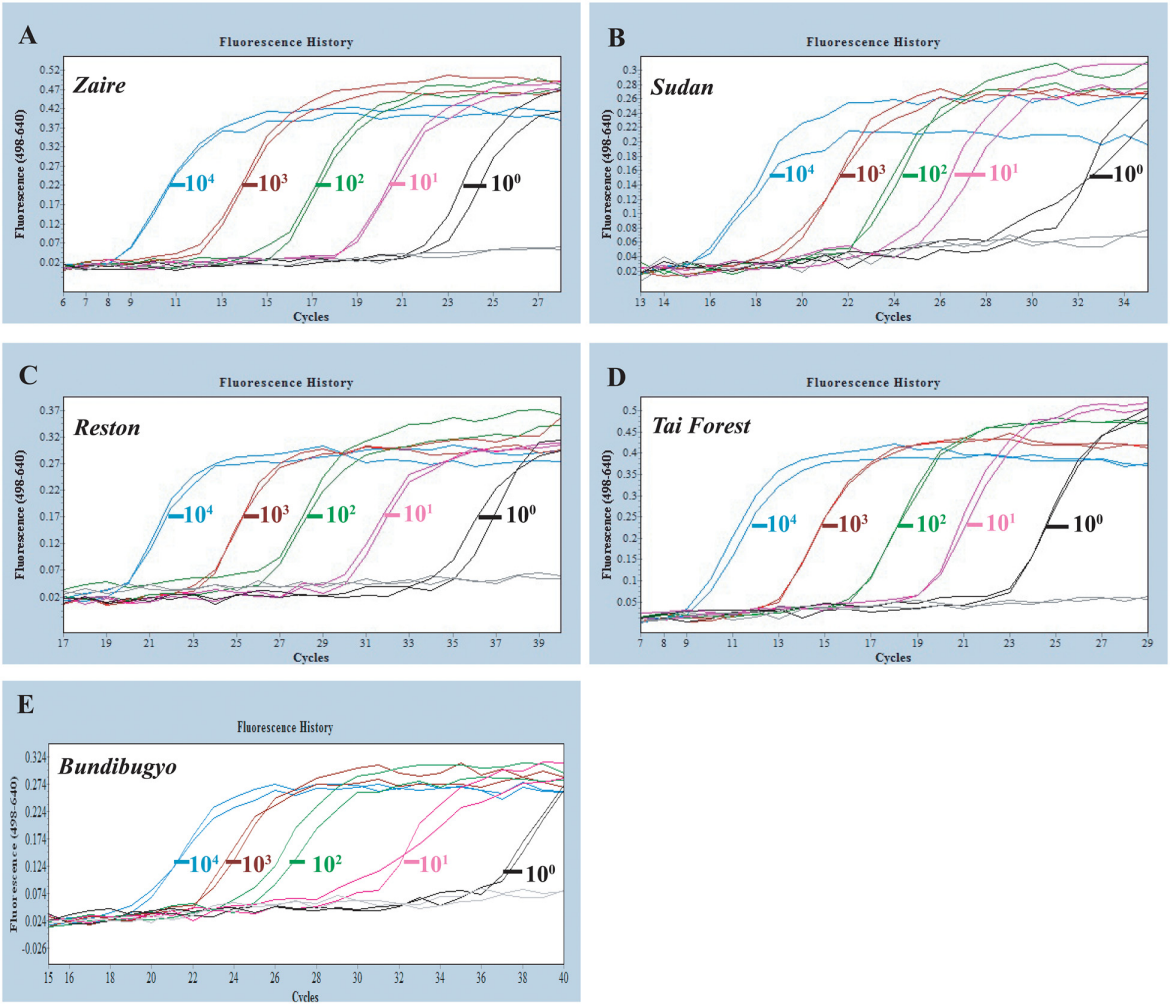
PCR amplification curves of serially diluted plasmids containing each of five *Ebolavirus* species. The plasmid quantitative standards (10^4^, 10^3^, 10^2^, 10^1^, 10^0^ /10μl) containing sequences of *Zaire ebolavirus* (**A**), *Sudan ebolavirus* (**B**), *Reston ebolavirus* (**C**), *Taï Forest ebolavirus* (**D**), *Bundibugyo ebolavirus* (**E**) and negative control were detected by the one-step reverse transcription FRET-PCR established in this study. The detection sensitivity was one copy per reaction system.

The BLASTN with the organism options of including and excluding *Ebolavirus* (taxid: 186536) was performed on each of the primers and probes to verify the specificity of the designed oligonucleotides we designed. Specificity of the one-step RT FRET-PCR was also evaluated by amplifying each of five plasmids containing portions of each *Ebolavirus* species (*Zaire ebolavirus*, *Sudan ebolavirus*, *Reston*, *ebolavirus Taï Forest ebolavirus* and *Bundibugyo ebolavirus*), and a mixture of the five plasmids (Fig 3). In addition, the following oligonucleotide polymers were synthesized (GenScript, Nanjing, China) to further test the sensitivity of the established PCR: 1) the first 100 bp of the PCR amplicon for EVD Zaire (contains forward primer and two probes); 2) the complimentary sequences of the first 100 bp of the PCR amplicon for EVD Zaire; 3) the 33–250 bp of the the PCR amplicon for EVD Zaire (contains two probes and five downstream primers).

**Fig 4:**
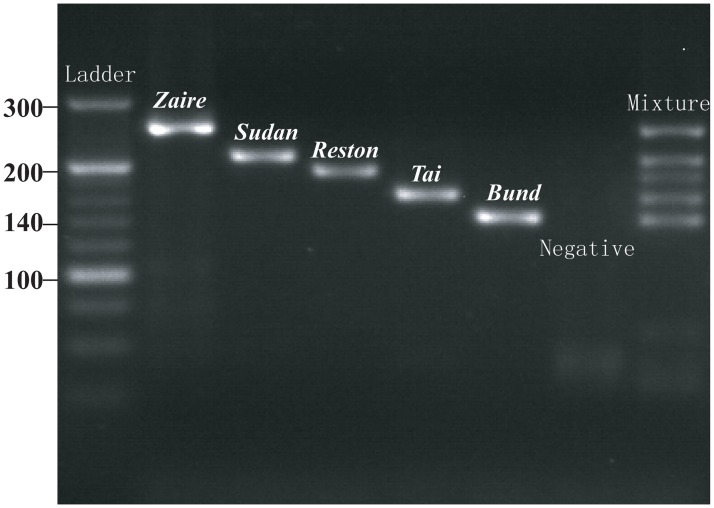
Discrimination of five *Ebolavirus* species by gel electrophoresis of PCR fragments. Plasmids of the five *Ebolavirus* species (*Zaire*, *Sudan*, *Reston*, *Taï Forest* and *Bundibugyo*), a mixture of all five, and negative controls were tested in the one-step FRET-PCR system established in this study. Amplicons obtained were electrophoresed through a 4% agarose gel and stained with SYBR Safe Gel stain (SYBR Safe DNA Gel Stain, Invitrogen, USA). The different sizes of the PCR products of each species (255 bp, 211 bp, 192 bp, 166 bp and 146 bp) were visualized alongside a 20bp DNA ladder (Thermo Scientific O’RangeRuler 20 bp DNA Ladder, Ready-to-use, Thermo Scientific, USA).

The specificity of the established PCR in this work was further tested on the nucleic acids of other pathogens causing hemorrhagic fever or similar clinical signs as Ebolavirus (Bunyavirus, Japanese encephalitis virus, Cuevavirus, Dengue virus, Yellow fever virus, Hanta virus, forest encephalitis virus, Lassa fever virus, Marburg virus,; Nucleic acids of these viruses were provided by the National Institute for Food and Drug Control, China Food and Drug Administration). The PCR products from the reactions with the plasmids of the individual *Ebolavirus* species and the mixture containing them all, were verified by electrophoresis through a 4% agarose gel (BIOWEST, Hong Kong, China) at 90 v for 90 min and stained with SYBR Safe DNA Gel Stain (Invitrogen, Carlsbad, USA) (Fig 4). A high concentration (4%) of BIOWEST Gel (gel strength≥750, gelling range around 36–39°C, and melting range around 87–89°C) ensures efficient differentiation of the small sizes of PCR fragments in this study.

#### Sensitivity of the one-step RT FRET-PCR

The sensitivity was determined using serially diluted plasmids (10^4^, 10^3^, 10^2^, 10^1^, 10^0^ copies of DNA/ 10 μl), transcribed *Ebolavirus* RNAs (10^5^, 10^4^, 10^3^, 10^2^, 10^1^, 10^0^ copies of RNA/ 10 μl) and *Ebolavirus* RNA-containing VLPs (10^5^, 10^4^, 10^3^, 10^2^, 10^1^, 10^0^ copies of RNA/ 10 μl). Also, human whole blood spiked with transcribed RNAs (10^6^, 10^5^, 10^4^, 10^3^, 10^2^, 10^1^ copies of RNA/tube with 50 μl whole blood) was amplified to determine the detection limit of the established one-step RT-PCR that could be expected with clinical samples.

## Results and Discussion

**Fig 5:**
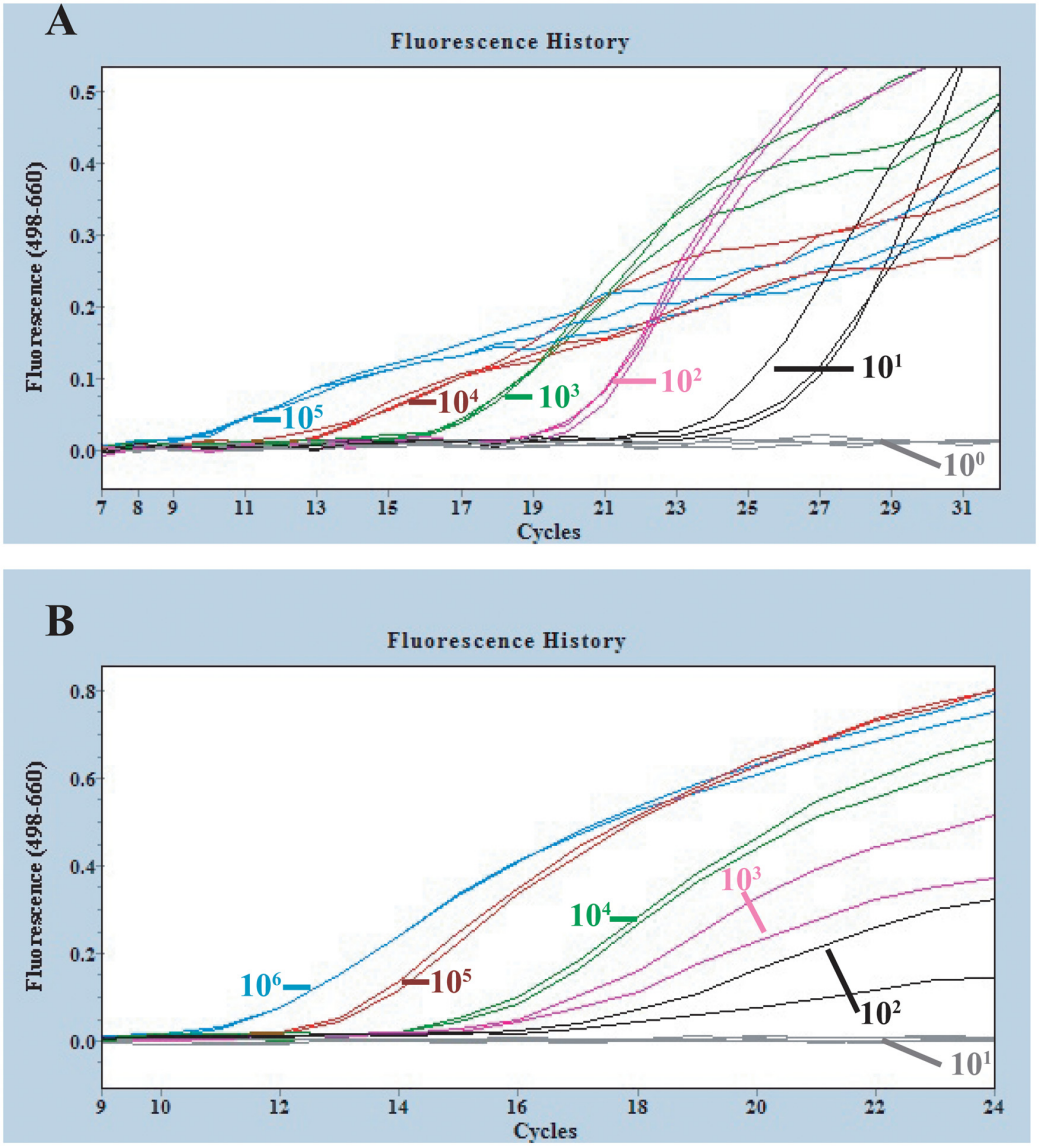
Amplification curves of reverse-transcription PCR on transcribed *Ebolavirus* RNAs and blood samples spiked with transcribed RNA. **A**: The different concentrations of transcribed *Zaire ebolavirus* RNA (10^5^, 10^4^, 10^3^, 10^2^, 10^1^, 10^0^ /10μl) were detected with the one-step reverse transcription FRET-PCR described in this study, and the detection limitation was 10 copies per reaction system. **B**: Serial concentrations of transcribed *Zaire ebolavirus* RNAs (10^6^, 10^5^, 10^4^, 10^3^, 10^2^, 10^1^/10μl) were spiked into human whole blood and were extracted with RNA purification kit, followed by the one-step reverse transcription FRET-PCR established in this study. The detection limitation was 100 RNA copies per reaction, equivalent to 320 copies of Zaire *Ebolavirus* in 1 ml whole blood. Triplicates (**A**) or duplicates (**B**) were performed for each concentration of tested *Ebolavirus* RNA.

Our one-step RT FRET-PCR was capable of detecting a single DNA copy and 10 RNA copies of all five *Ebolavirus* species (Figs 3 and 5). When whole blood spiked with transcribed *Ebolavirus* RNA and VLPs were tested, the detection limit was 100 copies of RNA per PCR reaction (Fig 5), equivalent to 320 copies of *Ebolavirus* in 1 ml whole blood. Triplicates were performed for each assay. The BLASTN results for each of the 8 oligonucleotides we used in the one-step RT FRET-PCR showed they were specific for the *Ebolavirus* species and did not cross-react with partial amplicons or other viruses in the family *Filoviridae*. No PCR amplification was observed with nucleic acids of eight types of pathogens which induce hemorrhagic fever or similar clinical signs as *Ebolavirus*.

**Fig 6:**
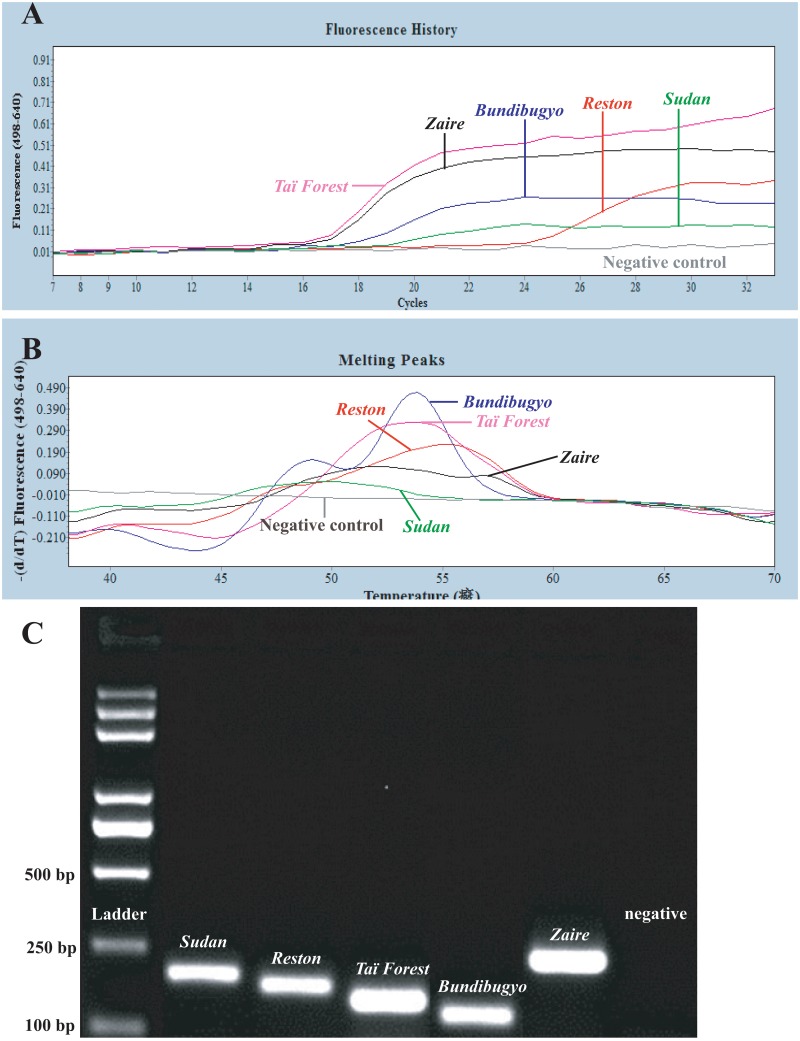
The sensitivity, melting curve analysis and gel electrophoresis of the one-step RT-FRET-PCR performed on *Ebolavirus* RNA-containing VLPs. The amplification curves of 100 copies of VLPs containing each of these five *Ebolavirus* species were run in the RT FRET PCR established in this study (**A**), followed by melting curves analysis (**B**) and gel electrophoresis (**C**). The Amplification curves of reverse-transcription PCR on serially diluted VLPs inoculated in blood sample showed similar sensitivity as in blood samples spiked with transcribed RNA (data not shown).

Due to the different levels of mismatches between the probes we designed and the PCR amplicons we generated from the different *Ebolavirus* species (Fig 1), melting curve analysis of the PCRs products showed unique and distinct *T*_m_ for each of the five *Ebolavirus* species (Fig 2): *Zaire*: flat dual peaks between 53.0°C and 56.9°C; *Sudan*: 51.6°C; *Reston*: flat dual peaks between 47.5°C and 54.9°C; *Taï Forest*: 52.8°C; *Bundibugyo*: dual peaks at 48.9°C and 53.5°C. The temperatures and shapes of the melting curves were consistent even with changes in the concentrations of *Ebolavirus* used in the reaction (Fig 2). Furthermore, the specific reverse primers we designed gave amplicons of different sizes for each of the five *Ebolavirus* species (*Zaire ebolavirus* 255bp; *Sudan ebolavirus* 211bp; *Reston ebolavirus* 192bp; *Taï Forest ebolavirus* 166bp; *Bundibugyo ebolavirus* 146bp) (Fig 4). The detection sensitivity, melting temperatures and amplicon sizes of each of these five *Ebolavirus* species were confirmed when the established RT-PCR was applied on *Ebolavirus* NP RNA-containing VLPs (Fig 6).

In the one-step RT-PCR established in this study, the combination of a single set of forward primer and probes with five different reverse primers gave very high sensitivity and differential ability. While the system detects all five *Ebolavirus* with highly sensitivity (1 copy of DNA, 10 copies of RNA, or 320 copies of RNA / 1 ml whole blood), the unique *T*_m_ distribution enables the convenient differentiation of the five *Ebolavirus* species (Fig 2). In laboratories that do not have real-time PCR machines and melting curve analysis, our primers can be used in standard PCRs and the five *Ebolavirus* species differentiated by the specific size of the amplicon identified for each species in gel electrophoresis (Fig 4).

While RNA templates as positive controls were not always available and safe for highly infective pathogens such as SARS virus and highly pathogenic avian influenza viruses, the VLPs equipped with specific RNA fragments have been widely used as stable, reliable and safe positive controls for RT-PCR [22–24]. The VLPs containing *Ebolavirus* NP RNA established in this work are adopted as standards to evaluate and certify *Ebolavirus*-related commercial diagnostic kits by National Institute for Food and Drug Control, Food and Drug Administration of China.

While simple PCR tends to provide high sensitivity but lacks ability to differentiate multiple targets simultaneously, multiplex PCR has the capacity to detect and differentiate multiple targets but tends to have a reduced sensitivity. Our study has established a novel highly-sensitive one-step RT FRET-PCR with one set of upstream primer /probes and five downstream primers that can rapidly identify and differentiate all five Ebolavirus species.
